# Improved YOLO-FastestV2 wheat spike detection model based on a multi-stage attention mechanism with a LightFPN detection head

**DOI:** 10.3389/fpls.2024.1411510

**Published:** 2024-06-19

**Authors:** Shunhao Qing, Zhaomei Qiu, Weili Wang, Fei Wang, Xin Jin, Jiangtao Ji, Long Zhao, Yi Shi

**Affiliations:** ^1^ College of Agricultural Equipment Engineering, Henan University of Science and Technology, Luoyang, Henan, China; ^2^ Science and Technology Innovation Center for Completed Set Equipment, Longmen Laboratory, Luoyang, China

**Keywords:** YOLO-FastestV2, wheat spike, efficient channel attention, Efficient Multi-Scale Attention, SimLightFPN

## Abstract

The number of wheat spikes has an important influence on wheat yield, and the rapid and accurate detection of wheat spike numbers is of great significance for wheat yield estimation and food security. Computer vision and machine learning have been widely studied as potential alternatives to human detection. However, models with high accuracy are computationally intensive and time consuming, and lightweight models tend to have lower precision. To address these concerns, YOLO-FastestV2 was selected as the base model for the comprehensive study and analysis of wheat sheaf detection. In this study, we constructed a wheat target detection dataset comprising 11,451 images and 496,974 bounding boxes. The dataset for this study was constructed based on the Global Wheat Detection Dataset and the Wheat Sheaf Detection Dataset, which was published by PP Flying Paddle. We selected three attention mechanisms, Large Separable Kernel Attention (LSKA), Efficient Channel Attention (ECA), and Efficient Multi-Scale Attention (EMA), to enhance the feature extraction capability of the backbone network and improve the accuracy of the underlying model. First, the attention mechanism was added after the base and output phases of the backbone network. Second, the attention mechanism that further improved the model accuracy after the base and output phases was selected to construct the model with a two-phase added attention mechanism. On the other hand, we constructed SimLightFPN to improve the model accuracy by introducing SimConv to improve the LightFPN module. The results of the study showed that the YOLO-FastestV2-SimLightFPN-ECA-EMA hybrid model, which incorporates the ECA attention mechanism in the base stage and introduces the EMA attention mechanism and the combination of SimLightFPN modules in the output stage, has the best overall performance. The accuracy of the model was P=83.91%, R=78.35%, AP= 81.52%, and F1 = 81.03%, and it ranked first in the GPI (0.84) in the overall evaluation. The research examines the deployment of wheat ear detection and counting models on devices with constrained resources, delivering novel solutions for the evolution of agricultural automation and precision agriculture.

## 1 Introduction

The yield and quality of wheat, one of the most important food crops in the world, are directly related to the sustainable development of agriculture as well as the guarantee of global food security (Misra et al., 2021). The number of wheat spikes is a key determinant of the number of wheat grains per unit area and the yield. Therefore, the number of wheat spikes is an important indicator of wheat yield (Slafer et al., 2014; Ferrante et al., 2017; Jin et al., 2017). The Measurement of the number of wheat spikes is important for fine-tuning the management of agricultural production, allocation of agricultural resources, and prediction of wheat yield (Pask, 2012; Zhou et al., 2021).

Currently, the methods used for the detection and counting of wheat spikes are categorized into manual counting, traditional machine learning detection and counting, and deep learning-based detection and counting methods. The manual counting method is cumbersome, labor-intensive, and subjective (Xiong et al., 2019). In traditional machine learning approaches, the detection and counting of wheat spikes usually relies on the selection of features, such as the shape, texture, and color of the ears, which are subsequently used to construct classification models for automated recognition and counting of wheat spikes (Zhao et al., 2014; Grillo et al., 2017; Ganeva et al., 2022). However, the method of manually extracting wheat features has the disadvantages of complex design, weak migration, and cumbersome manual design, which are less effective for applications in scenes with dense wheat spikes (Kamilaris and Prenafeta-Boldú, 2018; Zhang et al., 2020). Deep learning techniques have demonstrated excellent capabilities in the field of computer vision, particularly in the tasks of detecting and counting intensive objects, such as wheat spikes (Ye et al., 2023). These methods can automatically learn and extract key features from massive amounts of image data, thus effectively solving the complexity of manual feature extraction and the mobility problem in different scenarios using traditional methods (Sermanet et al., 2013). Therefore, deep learning provides a solution for the wheat spike counting task that is both efficient and accurate.

Many researchers have used deep-learning-based detection counting for detection and counting in agricultural-intensive scenarios (Aich et al., 2018; Wosner et al., 2021; Alkhudaydi and De La Iglesia, 2022). Common algorithms used for target detection in agriculture fall into two main categories: algorithms for single- and two-stage detection. One-stage algorithms, exemplified by Single Shot MultiBox Detector (SSD) (Liu et al., 2016), EfficientDet (Tan et al., 2020), and the YOLO family of algorithms (Redmon et al., 2016), directly classify and localize targets. Conversely, two-stage algorithms, such as SPP-Net (He et al., 2015), Mask R-CNN (He et al., 2017), and Faster R-CNN (Ren et al., 2015), first propose regions using RPN before refining classification and localization. Li et al. (2021) used the R-CNN method to detect, count, and analyze wheat spikes. The findings indicated that although the method demonstrated high recognition accuracy, it exhibited a slow detection speed and was unsuitable for deployment in a real-time detection device. Zhao et al. (2021) improved YOLOv5 by cleaning and enhancing data, adding a microscale detection layer, and adjusting the confidence loss function. They achieved an average accuracy of 94.1% in detecting wheat spikes in UAV images, surpassing the standard YOLOv5 by 10.8%. Li et al. (2022) used the Faster R-CNN model for image-based detection and metrology of the number of wheat spikes per unit area, demonstrating its practical application. Zang et al. (2022) introduced an efficient channel attention module (ECA) into the C3 module of the backbone structure of the YOLOv5s network model and simultaneously inserted a global attention mechanism module (GAM) between the neck and head structures. The results showed that the improved YOLOv5s method improved its applicability in complex field environments with better results. Wang et al. (2021) enhanced the wheat spike counting target detection model using Convolutional Block Attention Module (CBAM) in EfficientDet-D0, and the results of the study showed that the improved EfficientDet-D0 model achieves a counting accuracy of 94%, which is approximately 2% higher than that of the original model, and has a greater improvement in the occlusion problem. Wen et al. (2022) developed SpikeRetinaNet, a model consisting of a bi-directional feature pyramid network and a feature pyramid network, for detecting and counting the number of wheat spikes in field datasets for global wheat spike detection (GWHD). In general, among the various detection algorithms, single-stage algorithms have the advantages of higher speed and easier deployment in mobile segments, whereas two-stage algorithms, although they have higher accuracy and achieve better results, require large floating-point operations, and cannot achieve real-time recognition in edge devices with limited computational and storage resources (He et al., 2017; Ge et al., 2021). However, in practice, even single-stage algorithms often need to run on mobile devices that are more capable to ensure detection speed, which may not be sufficiently fast to meet real-time requirements on average mobile devices.

To achieve effective deployment and application of the model to mobile devices, researchers have developed a series of lightweight network architectures that can efficiently perform target detection tasks on mobile devices with limited resources (Zhang et al., 2021; Wang et al., 2022; Ma et al., 2023). Xu et al. (2020) introduced a hybrid YOLO V3-Lite lightweight model by incorporating residual blocks into YOLO-Lite. Using the Jetson AGX Xavier device, 43 FPS can be achieved for recognizing a 224 × 224 video image. Despite this, the number of parameters was still large at 20.5 MB. Tang et al. (2020) introduced a lightweight grape disease classification model using ShuffleNet as the backbone of the model and optimized it with the channel attention mechanism. The model achieved a recognition accuracy of 99.14% with only 1.1 M parameters. Yun et al. (2022) constructed a depthwise separable residual module by combining depthwise separable convolution and non-bottleneck residual modules. They replaced the VGG backbone network in the SSD network with this module and a depthwise separable convolutional structure to reduce the parameter count of the object detection model and enhance detection speed. Dong et al. (2024) improved the YOLOv5 algorithm by introducing Involution modules, Multi-Level Spatial Pyramid Pooling, Efficient Channel Attention (ECA) mechanism, and Content-Aware ReAssembly of Features upsampling method. As a result, the model’s parameter count decreased from 7.03 million to 6.09 million without sacrificing performance. Gao et al. (2021) designed a lightweight network backbone based on a novel spatial attention mechanism and migration learning to categorize garbage images and achieved good results in the garbage categorization task on the Huawei cloud platform with an accuracy rate of 96.17% and several floating-point operations of approximately 450M FLOPs. Dog-qiuqiu (2021) introduced the YOLO-FastestV2 model for real-time detection on mobile devices. The model aimed to substitute the YOLOV5 backbone with ShuffleNetV2 while mitigating the Feature Pyramid Network (LightFPN) structure. The parameter size of the model was only 237.55 kB. Good real-time detection can be achieved, even on embedded devices with limited computational resources. YOLO-FastestV2 has the advantages of having a small number of network parameters and low equipment requirements. Therefore, we selected YOLO-FastestV2 as the primary network for wheat spike detection.

When faced with the challenges of high density, severe occlusion, and overlap in wheat spike image detection, traditional detection methods are often difficult to accurately recognize, resulting in significant errors and missed detection problems. To solve these problems, an improved YOLO-FastestV2 target detection method was proposed in this study. In this study, three attention mechanism modules, Large Separable Kernel Attention (LSKA), Efficient Channel Attention (ECA), and Efficient Multi-Scale Attention (EMA), are introduced to enhance the feature extraction capability of the backbone network, effectively filter out irrelevant information, and improve the accuracy of wheat spike detection. For the LightFPN module of the detection head in YOLO-FastestV2, SimConv was used to replace the traditional Conv layer to further improve the accuracy of the model. Subsequently, a hybrid-enhanced wheat spike detection model was constructed by combining the improvements in the backbone network with the detection head. In addition, this article provides a comprehensive evaluation of the accuracy, size, and computational complexity of the model, with the aim of finding a model that strikes a balance between performance and resource usage.

## 2 Materials and methods

### 2.1 Dataset construction

**Figure 1:**
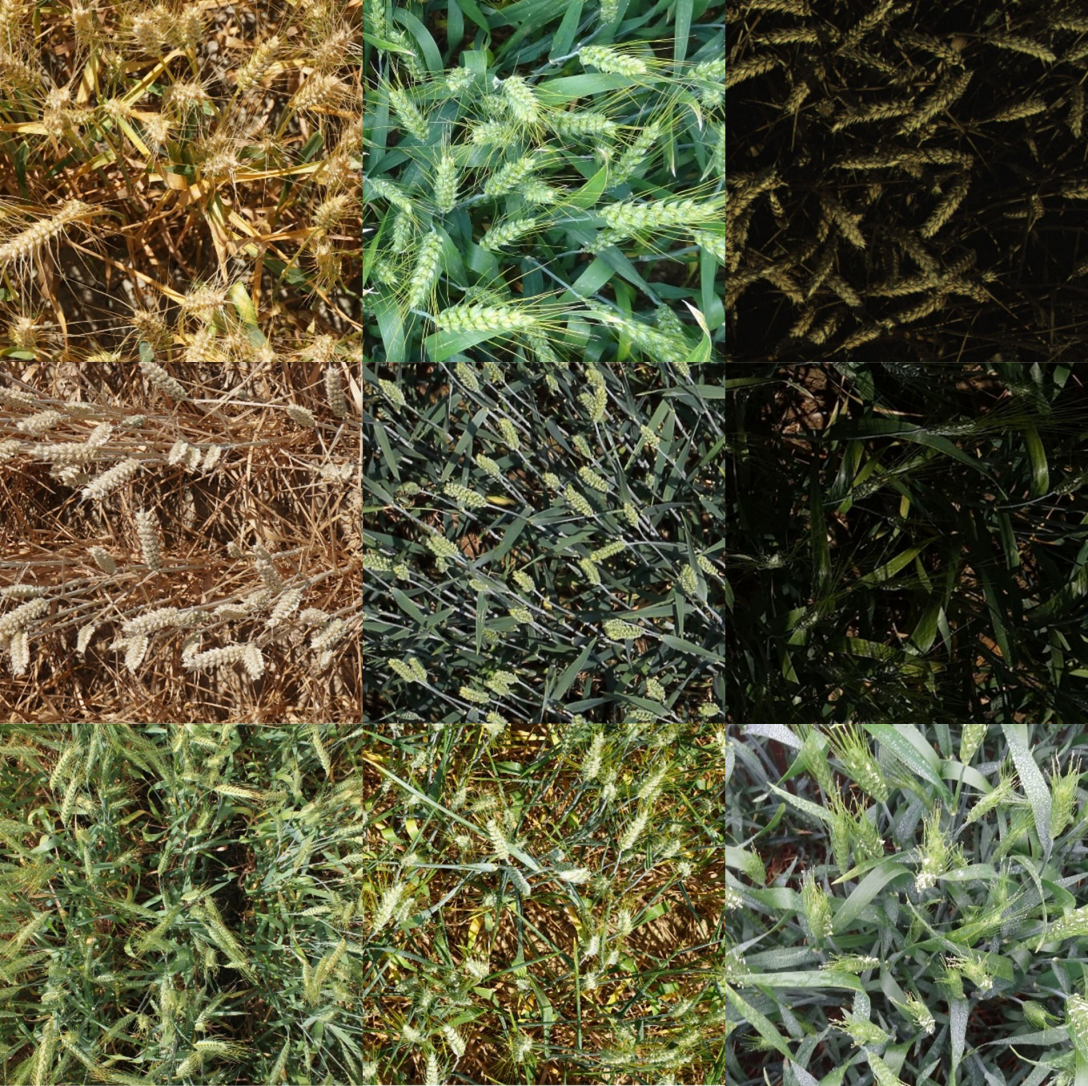
Example of the wheat spike dataset.

The wheat spike detection dataset utilized in this study is sourced from a variety of datasets, including the Global Wheat Detection 2020 dataset available at Kaggle, the Global Wheat Detection 2021 dataset found at Global Wheat, and a custom dataset provided by PP Flying Paddle, accessible at Baidu AI Studio. The Global Wheat Detection dataset came from 11 countries/regions and covered 44 unique measurement sessions (David et al., 2021). A measurement session is a set of images acquired using a specific sensor at the same location within a coherent timestamp (typically a few hours). This study reorganized three datasets, deleted blank and duplicate images, and retained 11,451 images. We reorganized all wheat spike categories and named all categories “wheat-spike”. We retained only one unique category label, wheat spikes, to enhance the generalization performance of the training model, capture the common specific points of each type of wheat spike, and accommodate the features of the different categories of wheat spikes. In this study, the dataset was divided into a training set, validation set, and test set in the ratio of 8:1:1. The number of images in the training set was 9160 and the number of detection frames was 397411. The number of images in the validation set was 1145 and the number of detection frames was 50137. The number of images in the test set was 1146 and the number of detection frames was 49426. We selected several pictures of wheat spikes of different maturities and under different light conditions, as shown in **Figure 1**.

### 2.2 YOLO-FastestV2 network architecture

**Figure 2:**
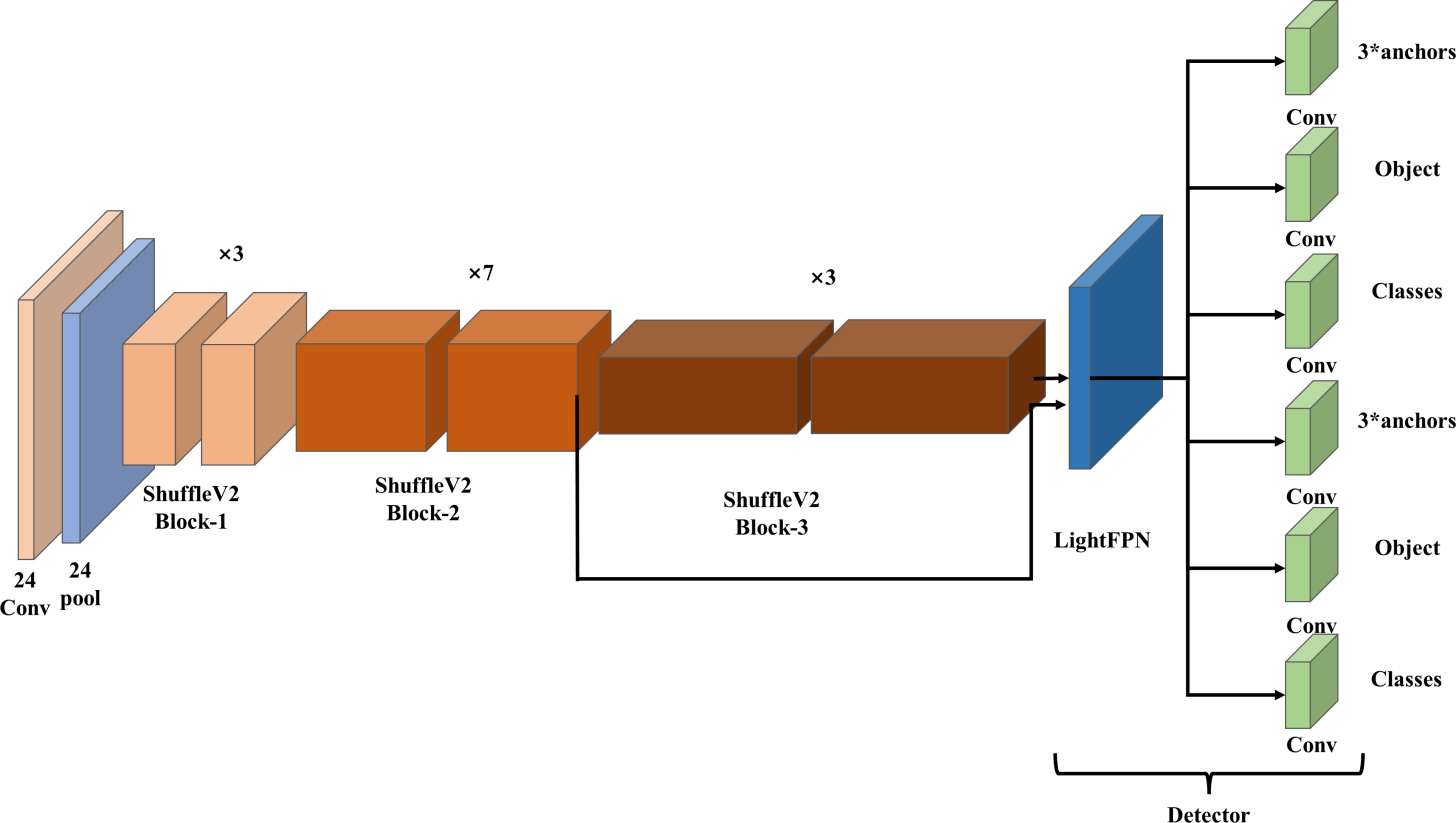
Diagram of YOLO-FastestV2 network structure.

**Figure 3:**
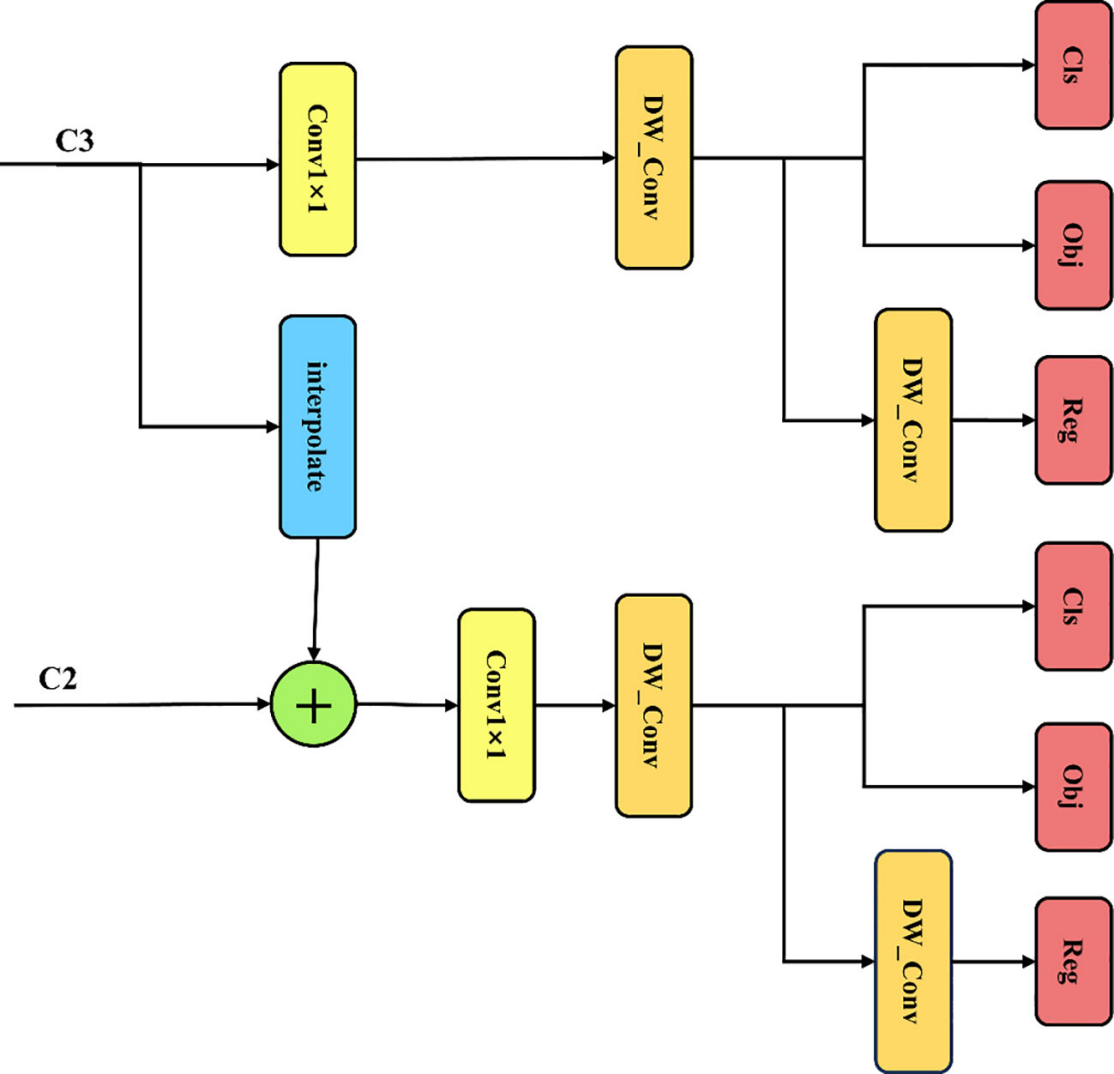
LightFPN module structure.

YOLO-FastestV2 consists of three parts: input layer, backbone network, and detection header. The main structure of the system is shown in **Figure 2**. The input layer consisted of a 24 × 24 convolutional module and a pooling layer. The backbone network uses the ShuffleNetV2 (Chen et al., 2022) network as the model for backbone feature extraction, which reduces the memory access cost, improves speed, and reduces model weight. The detection header part is used by the LightFPN module and Yolov5 anchor-matching mechanism to predict the actual detection frame. The structure of the LightFPN module is shown in **Figure 3**. Using 1 × 1 convolution and Depth Separable Convolution (DWConv) techniques, the LightFPN effectively reduces the computation and number of parameters of the model, making it more efficient to run on resource-constrained devices. LightFPN integrates the feature maps of different layers through up-sampling and down-sampling operations to capture the information of the image at different scales. LightFPN uses up-sampling and down-sampling techniques to effectively fuse feature maps of different resolutions to capture image information at different scales. Furthermore, using DWConv modules and hopping connections, LightFPN significantly improves the representation of the features. The feature map of Yolo-Fastest V2 can be decoupled into three different feature maps. The foreground context classification and detection classes use the same network branches and share the parameters to enable lightweight target detection.

### 2.3 Attention mechanism module

The attention mechanism plays a key role in the field of deep learning by weighting the input feature maps, reinforcing important features, and suppressing unimportant ones, allowing the network to focus better on the target region and improve the representation of features (Qiu et al., 2020). In this study, we used three different attentional mechanisms (ECA, LSKA, and EMA) to improve the YOLO-FastestV2 model to improve the detection accuracy of the model.

#### 2.3.1 ECA attention module

**Figure 4:**
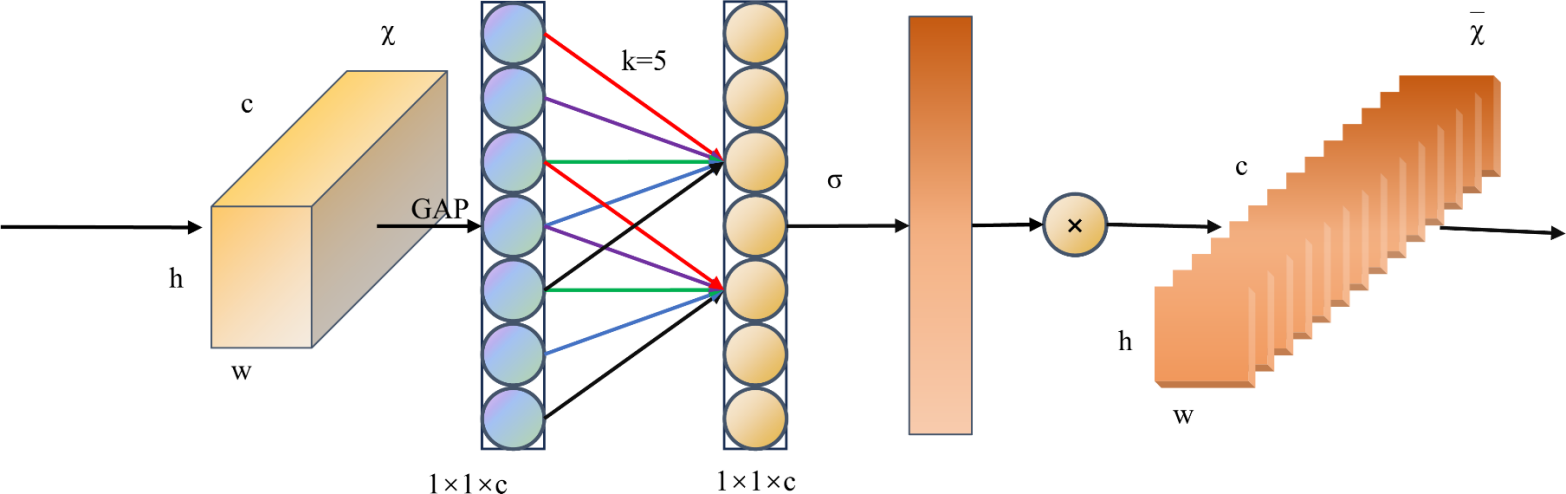
Structure of the ECA Attention Mechanism Module.


The ECA attention mechanism is an attention model designed to enhance the capacity of neural networks to model image features (Wang et al., 2020). At first, ECA abandons the dimensionality reduction operation in the SE module and maintains the direct correlation of the channels by maintaining the integrity of the information through 1 × 1 convolution. Secondly, ECA employs one-dimensional convolution to enhance local interactions between channels, which not only reduces information loss due to dimensionality reduction, but also allows the network to more fully utilize spatio-temporal information. In addition, the convolutional kernel size of ECA can be adaptively adjusted according to the number of channels and the depth of the network, providing flexibility to capture different ranges of channel dependencies. These features work together to enhance the performance of the model. The main structure of the ECA attention mechanism is illustrated in **Figure 4**. ECA first performs global average pooling (GAP) on the acquired feature maps, followed by application of a one-dimensional convolution operation with kernel size k. In this process, the weights of each channel are computed using a sigmoid activation function, the expression of which is shown in Equation 1.

(1) $$\omega =\sigma (C1D_{k}(y))$$

where $1D_{k}$ denotes a one-dimensional convolution with a convolution kernel size k. Kernel size k determines the range of the channel interaction and is related to the channel dimension, which denotes the visible channel function. In general, it is believed that the larger the channel size, the stronger are the long-term interactions, whereas the smaller the channel size, the stronger are the short-term interactions. In ECA, an adaptive approach is used to determine the size of the nucleus. Finally, the weights are multiplied by the corresponding elements of the original input feature map to obtain the final output feature map.

#### 2.3.2 LSKA attention mechanisms

**Figure 5:**
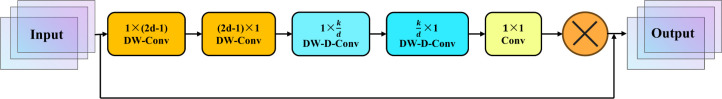
Structure of the LSKA attention mechanism.

The principle of the LSKA is an attention mechanism proposed in response to the problems faced by traditional Large Kernel Attention (LKA) when dealing with large convolutional kernels, such as high computational and memory requirements (Lau et al., 2024). The LSKA attention mechanism decomposes the two-dimensional convolutional kernels of the deep convolutional layer into cascading horizontal and vertical one-dimensional kernels, significantly reducing the number of parameters and computational complexity of the model. The LSKA maintains similar performance to the original LKA, effectively capturing key features of the image such as edges, texture and shape. In addition, the design of LSKA allows the use of large convolutional kernels directly in the attention module without the need for additional blocks, enhancing the model’s ability to model long distance dependencies of the input image. While reducing computational and memory requirements, LSKA improves the performance of vision tasks and enhances the robustness of the model to image perturbations. The LSKA attention structure is shown in **Figure 5**. The core of LSKA involves decomposing a conventional 2D convolutional kernel into two 1D convolutional kernels. First, it decomposes a large 2D kernel, horizontally and vertically, into two 1D kernels. This decomposition drastically reduces the number of parameters and computational complexity. Despite the decomposition and concatenation strategies, the performance of LSKA is similar to that of the original LKA. This means that the LSKA is effective in capturing important information when dealing with the key features of an image, such as edges, textures, and shapes.

#### 2.3.3 EMA attention mechanism

**Figure 6:**
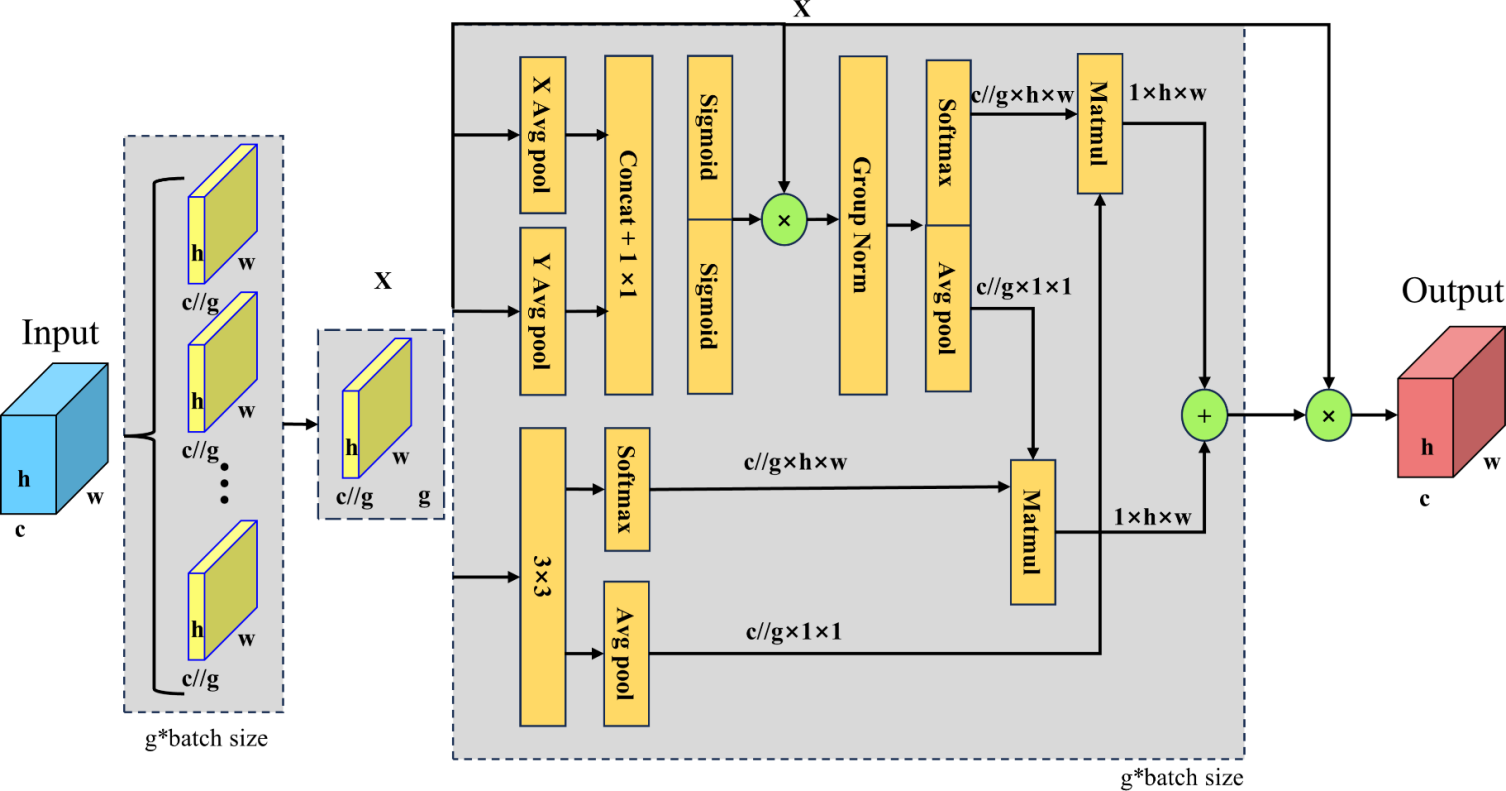
Structure of EMA attention mechanism.

EMA is an efficient multi-scale attention module based on cross-spatial learning designed to enhance the performance of deep learning models in computer vision tasks (Ouyang et al., 2023). The EMA attention mechanism captures multi-scale features through parallel 1x1 and 3x3 convolutional branches, where the 1x1 branch encodes channel attention using one-dimensional global average pooling, while the 3x3 branch is used to capture more detailed multi-scale spatial features. To enhance the feature representation through cross-spatial information aggregation while avoiding information loss through dimensionality reduction operations, EMA maintains direct correlation between channels. In addition, EMA reduces computational overhead by reshaping some of the channels into bulk dimensions, while enhancing the capacity of the model to perceive the global context of the image by encoding global spatial information. The main structure is illustrated in **Figure 6**. The EMA module divides the input feature map into subfunctions for each channel dimension. The EMA module uses three parallel routes to extract attention weight descriptors: two in the 1 × 1 branch and one in the 3 × 3 branch. The 1 × 1 branch uses one-dimensional global average pooling to encode channel attention in both spatial directions. The 3 × 3 branch uses a single 3 × 3 kernel to capture the multi-scale feature representations. The EMA module also passes the original input elements through a 3 × 3 branch to expand the feature space. The EMA module uses cross-spatial information aggregation to enrich the feature aggregation. The EMA module encodes global spatial information using two-dimensional global average pooling in 1 × 1 and 3 × 3 branches. The EMA module encodes global spatial information by applying two-dimensional global average pooling in 1 × 1 and 3 × 3 branches, generates spatial attention maps using matrix dot product operations, and then aggregates the output feature maps within each group to compute spatial attention weights.

#### 2.3.4 SimConv

SimConv, an innovative module within the architecture of Convolutional Neural Networks (CNNs), leverages the concept of similarity among image blocks to steer the convolution process. This strategic approach is designed to elevate the caliber of feature representation while concurrently boosting the network’s operational efficiency. The module’s underlying philosophy hinges on the discernment of representative and uncertain areas within the input feature map, followed by the application of a tailored processing strategy. This not only preserves the integrity of the feature set but also leads to a pronounced reduction in computational demands and a streamlined parameter count. The computational procedure of SimConv can be succinctly outlined as follows:


(2) y=ReLU(BN(x·ω))

In Equation 2, *x* denotes the input feature map, *www* symbolizes the convolution kernel, BN refers to Batch Normalization, and ReLU is indicative of the Rectified Linear Unit activation function. The forward_fuse technique optimizes the process by integrating Batch Normalization and the ReLU activation function directly into the convolution operation, thus achieving a decrease in both computational overhead and memory usage:


(3) y=ReLU(x·ω)

### 2.4 Evaluation of indicators

In this study, Precision (P), Recall (R), Average Precision (AP), and Mean Average Precision (mAP) were used as evaluation metrics to compare the effectiveness of different wheat spike detection models. where P is the probability of correctly detecting the target among all the detected targets, R is the probability of correctly recognizing the target among all the positive samples, and AP is the average value of the detector for each R case, which corresponds to the area under the PR curve. The mAP is the average of the averages computed for the APs of various categories, which summarizes the APs in terms of the category dimension to measure the performance of the multi-category target detection task. They are formulated as shown in **Equations 3**–**7**.


(4) $$P=\frac{TP}{TP+FP}$$

(5) $$R=\frac{TP}{TP+FN}$$

(6) $$AP=\frac{\sum P_{r_{i}}}{\sum r}$$

(7) $$mAP=\frac{AP}{num\_classes}$$

Where TP denotes that the predicted value is a positive sample as well as the true value. FP denotes that the predicted value is a positive sample and the true value is a negative sample. FN denotes that the predicted value is a negative sample, the true value is a positive sample, and num_classes denotes the number of classes. The ${\text{P}}_{{\text{r}}_{\text{i}}}$ denotes the P-value corresponding to r - i on the PR curve, ∑r=1 .

In this study, a suite of evaluation metrics was used to assess the accuracy of the model. Although these enhancement strategies have generally improved the precision of the model, they have also led to an increase in Giga Floating Point Operations per Second (GFLOPs) and Params, which may elevate the hardware requirements for the model. To comprehensively assess the performance of the improved model, we introduced the global evaluation index (GPI) as a comprehensive evaluation index, as shown in Equation 8.


(8) $$GPI=\sum_{j=1}^{4}\alpha_{j}\left(g_{j}-y_{j}\right)$$

where $g_{j}$ are MAP@0.5, MAP@0.5:0.95, GFLOPs, and Params, $y_{j}$ are the medians of the corresponding parameters. When $g_{j}$ is MAP@0.5, $\alpha_{j}$ takes the value 0.7 and when $g_{j}$ is MAP@0.5:0.95, $\alpha_{j}$ is 0.2; otherwise $\alpha_{j}$ is -0.05. MAP@0.5 is the result of calculating the AP of all images in each category when IoU is set to 0.5, and then averaged across all categories. mAP@.5:.95 indicates mean mAP at different IoU thresholds (from 0.5 to 0.95 in steps of 0.05) (0.5, 0.55, 0.6, 0.65, 0.7, 0.75, 0.8, 0.85, 0.9, and 0.95). At the same time, because the network we selected was lightweight, we gave GFLOPs and Params a smaller share in the overall evaluation. The higher the GPI value or the higher the ranking, the better is the performance of the model in terms of accuracy and the combined requirements of the device.

## 3 Results and discussion

### 3.1 Experimental platforms

In this study, the experimental platform we used was a desktop computer configured with a 10th generation Intel Core i5 processor with 16 GB of memory (RAM) and an NVIDIA GeForce RTX 2060 graphics card with 6 GB of video memory. The operating system was Windows 10 Professional and CUDA version 11.8. For programming, we used two tools, Python 3.10 and PyTorch 2.1.1.

### 3.2 Impact of inclusion of a single-stage attention mechanism on the model

**Figure 7:**
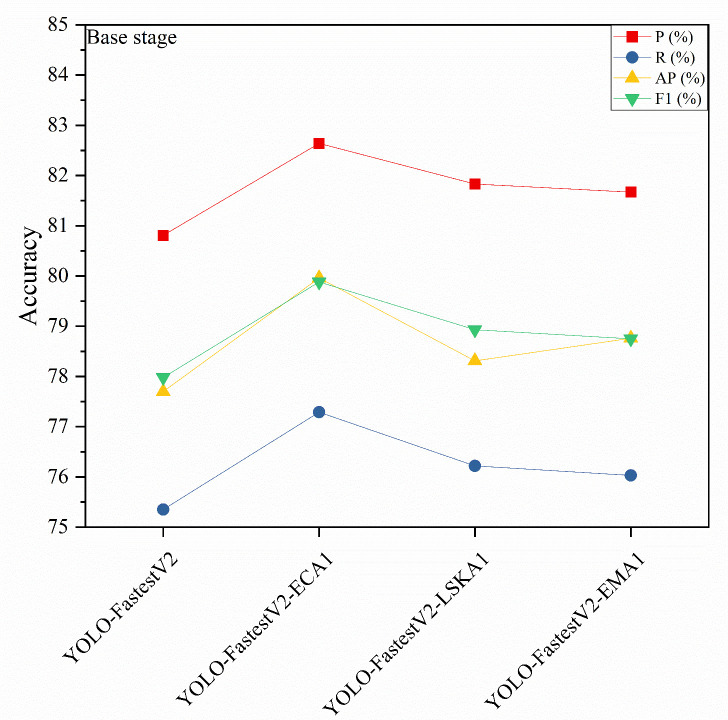
Model accuracy added by base stage attention mechanism.

**Figure 8:**
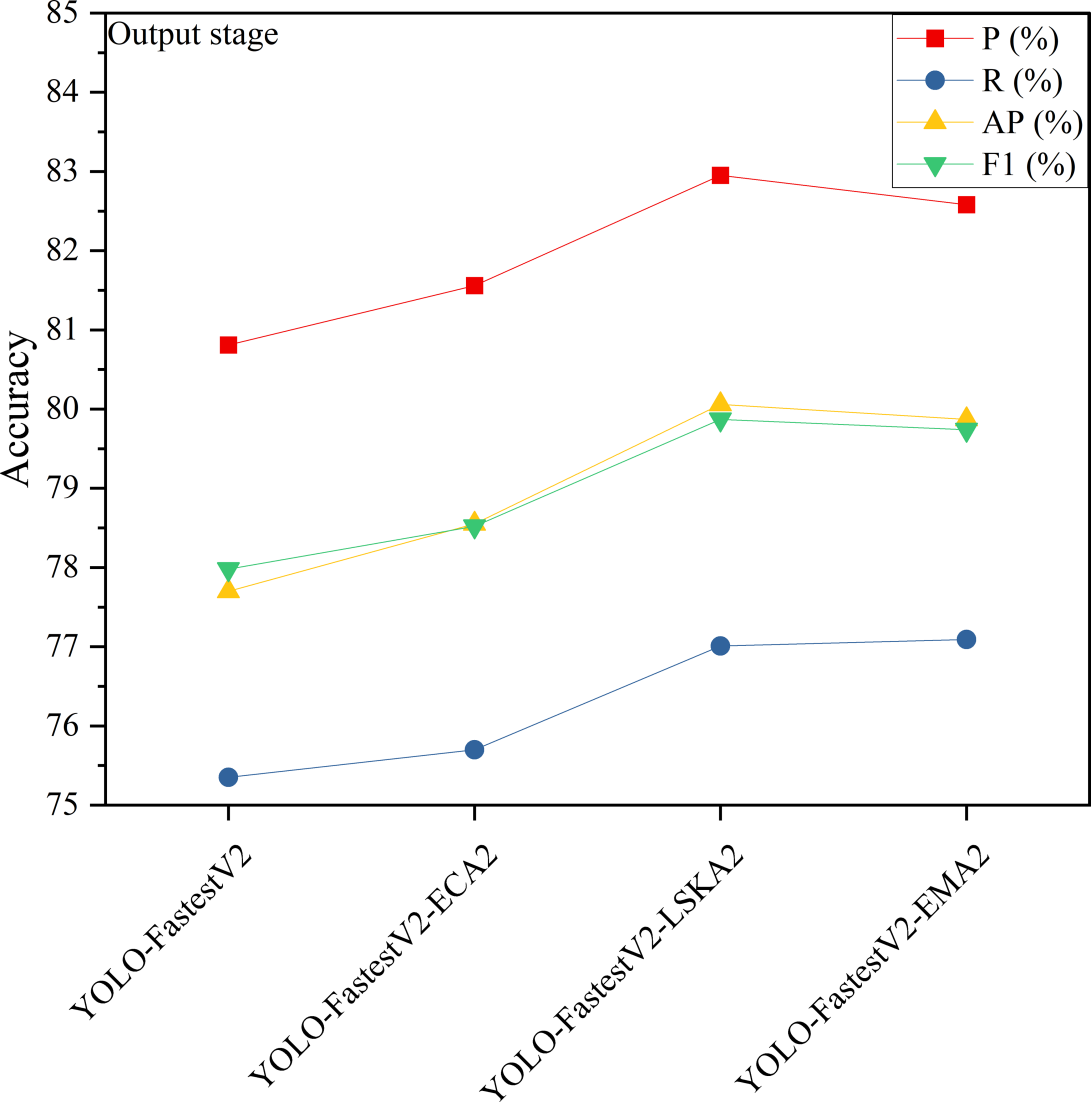
Model accuracy added by output stage attention mechanism.

In the YOLO-FastestV2 model, the backbone network uses ShuffleNetV2 architecture, which is divided into three main phases: foundation, transition, and output (Zhang et al., 2023). The outputs of the transition and output phases were fed into the subsequent LightFPN modules. Considering that different attention mechanisms have different focuses on feature extraction and selection, this study introduced an attention mechanism module after the base and output phases to enhance the feature extraction capability of the backbone network. We denoted the attention mechanism added after the foundation stage as 1 and the attention mechanism added after the output stage as 2. The modeling accuracy of the Attention Mechanism module, which was added separately after the different stages, is presented in **Figures 7** and **8**.

As shown in **Figure 7**, the accuracy of the YOLO-FastestV2 model is P=80.81%, R=75.34%, AP=77.70%, and F1 = 77.98%. Among the models with the three attention modules added in the base stage, YOLO-FastestV2-ECA1, with the addition of the ECA attention mechanism, exhibited the highest accuracy improvement with models with P=82.64%, R=77.29%, AP=79.96%, and F1 = 79.88%. YOLO-FastestV2-LSKA1 with the addition of the LSKA attention mechanism exhibited a higher accuracy improvement (P=81.83%, R=76.22%, AP=79.31%, and F1 = 78.93%). The Yolo fastestV2_LSKA1 model showed higher accuracy gains (P=81.83%, R=76.22%, AP=78.31%, and F1 = 78.93%). The YOLO-FastestV2-EMA1 model, with the addition of attention to the EMA attentional mechanism, had smaller accuracy gains (P=81.67%, R=76.03%, AP= 78.76%, and F1 = 78.75%). As shown in **Figure 8**, among the models with the three attention modules added after the output stage, the YOLO-FastestV2-LSKA2 model with the addition of the LSKA attention mechanism module achieved the highest accuracy with a model accuracy of P=82.95%, R=77.01%, AP=80.06%, and F1 = 79.87%. This was followed by the YOLO-FastestV2-EMA2 model, which exhibited a greater increase in accuracy with the addition of the EMA attention mechanism module (P=82.58%, R=77.09%, AP=79.87%, and 79.74%). Lastly, the YOLO-FastestV2-ECA2 model, with the addition of the ECA attention mechanism module, exhibited the lowest accuracy improvement with P=81.56%, R=75.70%, AP=78.55%, and F1 = 78.52%.

The ECA attention mechanism improves the performance of the network by adaptively selecting a one-dimensional convolutional kernel size to adjust the coverage of local cross-channel interactions to avoid losing the target feature information during the dimensionality reduction process (Guo et al., 2021). The LSKA attention mechanism combines local self-attention with a large convolutional kernel to enable the model to focus on specific regions in the image, thereby improving its ability to recognize key features and local structures in the image (Lau et al., 2024). The EMA attention mechanism enhances the feature recognition capability of the model by reshaping some of the channel dimensions into batch dimensions and constructing local cross-channel interactions in each parallel sub-network, as well as increasing the interaction of the channel information by fusing the output feature maps of the two parallel sub-networks through a cross-space learning approach (Sun et al., 2024). The model with the ECA attention mechanism added at the base stage (YOLO-FastestV2-ECA1) showed the highest accuracy improvement, suggesting that ECA is effective in enhancing the model’s recognition of the target features in the image, especially in the early stages of the model. LSKA showed a high accuracy improvement both in the base stage (YOLO-FastestV2-LSKA1) and after the output stage (YOLO-FastestV2-LSKA2). This indicated that LSKA is effective in enhancing the performance of the model at different stages, particularly in recognizing local features in the image. The model with the EMA attention mechanism added after the output stage (YOLO-FastestV2-EMA2) exhibited the higher accuracy, whereas the model with the EMA added at the base stage (YOLO-FastestV2-EMA1) exhibited a smaller accuracy gain. This suggests that EMA is more effective in the later stages of the model because it may require more contextual information to optimize feature fusion. Although the three attentional mechanisms improved the accuracy of the model to different degrees, the same attentional mechanism had different effects on the accuracy of the model at different stages of the network.

### 3.3 Impact of inclusion of two-stage attention mechanism on modeling

**Figure 9:**
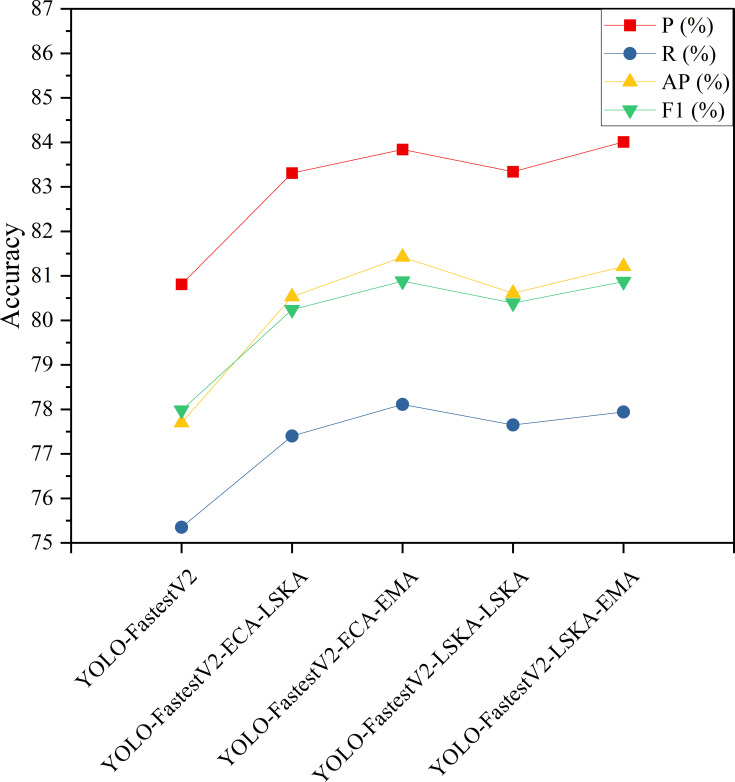
Modeling accuracy of two-stage increasing attention mechanism.

To further enhance the extraction performance of target features of the backbone network, the attention mechanisms that have higher performance enhancement of the model at different stages were selected in this study to be used in the construction of the backbone network with hybrid attention mechanisms to enhance the performance of the model. Among the results of the model enhancement by the single-stage attention mechanism modules, ECA and LSKA added after the base stage improved the accuracy of the model, and the addition of LSKA and EMA attention mechanism modules after the output stage improved the accuracy of the model. The accuracy results of the two-stage addition of the attention mechanism model are listed in **Figure 9**.

As can be observed from **Figures 7**, **8**, the experimental results of combining the attention mechanisms showed that the combination of different attention mechanisms improved the accuracy of the model. From **Figure 9**, it can be observed that among the four models with combined attention mechanisms, the YOLO-FastestV2-ECA-EMA model with the combination of ECA and EMA attention mechanisms had the highest overall accuracy improvement (P=83.84%, R=78.11%, AP=81.42%, and F1 = 80.88%), and the LSKA and EMA attention mechanisms with the combination of YOLO-FastestV2-LSKA-EMA model had a higher accuracy improvement with a model accuracy of P=84.01%, R=77.94%, AP=81.21%, and F1 = 80.87%. Third, we used the YOLO-FastestV2-LSKA-LSKA model with the addition of the LSKA attentional mechanism in both the stages (P=83.34%, R=77.65%, AP=80.61%, and F1 = 80.39%). Finally, the YOLO-FastestV2-ECA-LSKA model with a combination of the two attentional mechanisms, ECA and LSKA, exhibited the lowest accuracy improvements of P=83.31%, R=77.40%, AP=80.53%, and F1 = 80.24%.

The YOLO-FastestV2-ECA-EMA model performed the best in terms of overall accuracy, which may be because ECA optimized the feature representation in the early stage, whereas EMA further refined the multi-scale information of the features in the later stage. The combination of both may have formed a complementary approach, which makes the model effective in extracting and utilizing features in different stages. The higher accuracy improvement of the YOLO-FastestV2-LSKA-EMA model may be because LSKA performed effective local and global structure extraction of the features in the early stages, whereas EMA further refined these features in the later stages, and the combination of the two may similarly form a complementary effect. The YOLO-FastestV2-LSKA-LSKA model ranked third in terms of improvement in accuracy. This suggests that using LSKA in both phases may offer an advantage in the depth of feature extraction, but may not have the benefit of ECA or EMA in multi-scale feature capture. The YOLO-FastestV2-ECA-LSKA model showed the smallest improvement in accuracy, possibly owing to some overlap between the mechanisms. This overlap may cause the network focus to overly prioritize localized information, leading to limited improvement in model accuracy.

### 3.4 Impact of improved LightFPN on modeling

In the YOLO-FastestV2 algorithm, the LightFPN module was used in the detection head section to process the output results of the backbone network. Despite its lightweight design, the LightFPN encounters challenges in wheat spike detection, particularly in scenarios with uniform backgrounds and complex scene dynamics. To enhance detection accuracy in these conditions, we integrated the SimConv module to replace the traditional Conv module within the LightFPN architecture, resulting in the SimLightFPN. The SimConv module primarily consists of Conv, BatchNorm, and ReLU to accelerate the convergence of the model, which allows the direct application of convolution and activation functions without batch normalization by providing the forward_fuse method (Hu and Zhu, 2023). The accuracy of the improved model is shown in **Table 1**.

**Table 1:** Improved SimLightFPN model with original model accuracy.

Model	P (%)	R (%)	AP (%)	F1 (%)
YOLO-FastestV2	80.81	75.35	77.70	77.98
YOLO-FastestV2-SimLightFPN	83.65	77.54	80.51	80.48

As can be observed from **Table 1**, the accuracy of the improved model was P=83.65%, R=77.54%, AP=80.51%, and F1 = 80.48%, which is an increase of 2.84% for P, 2.19% for R, 2.81% for AP, and 2.5% for F1, compared with the original model. SimLightFPN, while retaining the core advantages of LightFPN, optimized the forward propagation process of the feature fusion network through the introduction of SimConv, thereby significantly enhancing the performance of the algorithm in handling the feature fusion task (Yue et al., 2023). The results suggest that SimConv may be more efficient for feature extraction and fusion, particularly when dealing with similar backgrounds and complex scenes in whelk detection.

### 3.5 Multi-stage improved hybrid models

To further enhance the performance of the model, two models, YOLO-FastestV2-ECA-EMA and YOLO-FastestV2-LSKA-EMA, which have higher accuracy for the two-stage addition of the attention mechanism, were selected in this study and combined with the improved SimLightFPN module to construct a multi-stage improved hybrid model. The accuracies of the improved hybrid models are listed in **Table 2**.

**Table 2:** Accuracy of the multi-stage improved hybrid model.

Model	P (%)	R (%)	AP (%)	F1 (%)
YOLO-FastestV2	80.81	75.35	77.70	77.98
YOLO-FastestV2-SimLightFPN-LSKA-EMA	84.28	78.37	81.85	81.22
YOLO-FastestV2-SimLightFPN-ECA-EMA	83.91	78.35	81.52	81.03

As can be observed in **Table 2**, in the multi-stage improved hybrid model, the YOLO-FastestV2-SimLightFPN-LSKA-EMA model showed the highest accuracy improvement (P=84.28%, R=78.37%, AP=81.85%, and F1 = 81.22%) and the YOLO-FastestV2-SimLightFPN- ECA-EMA showed a higher accuracy improvement with models with P=83.91%, R=78.35%, AP=81.52%, and F1 = 81.03%. The YOLO-FastestV2-SimLightFPN-LSKA-EMA model performed the best in terms of accuracy improvement, which may be attributed to the advantages of the combination of the LSKA and EMA attention mechanisms in terms of an in-depth understanding of features and multi-scale information extraction. Combined with the feature fusion capability of SimLightFPN, this enabled the model to capture and analyze features at different depths better in the wheat spike detection task. Although the accuracy improvement of the YOLO-FastestV2-SimLightFPN-ECA-EMA model was slightly lower than that of the YOLO-FastestV2-SimLightFPN-LSKA-EMA model, it still exhibited high-performance improvement. The results show that the combination of SimLightFPN and the two-stage add-attention mechanism can effectively fuse and analyze the features of different depths to improve the accuracy of the model.

### 3.6 Comprehensive evaluation

The ranked results of the GPI for all models are listed in **Table 3**.

**Table 3:** Value of GPI for all models.

Model	mAP@0.5 (%)	mAP@.5:.95 (%)	GFLOPs	Params	GPI	Rank
YOLO-FastestV2	67.57	55.10	0.38	0.24	0.00	14
YOLO-FastestV2-ECA1	70.41	60.00	0.38	0.24	0.60	6
YOLO-FastestV2-ECA2	67.78	55.12	0.38	0.24	0.03	13
YOLO-FastestV2-LSKA1	67.97	57.03	0.4	0.24	0.09	12
YOLO-FastestV2-LSKA2	69.93	58.90	0.39	0.28	0.42	10
YOLO-FastestV2-EMA1	68.58	56.63	0.4	0.24	0.17	11
YOLO-FastestV2-EMA2	69.77	58.76	0.39	0.24	0.44	9
YOLO-FastestV2-ECA-LSKA	70.23	59.26	0.39	0.28	0.48	8
YOLO-FastestV2-ECA-EMA	71.58	61.41	0.39	0.24	0.81	3
YOLO-FastestV2-LSKA-LSKA	70.59	59.85	0.41	0.28	0.52	7
YOLO-FastestV2-LSKA-EMA	71.07	60.68	0.41	0.25	0.66	5
YOLO-FastestV2-SimLightFPN	70.75	60.66	0.38	0.24	0.67	4
YOLO-FastestV2-SimLightFPN-LSKA-EMA	72.00	61.71	0.41	0.25	0.84	2
YOLO-FastestV2-SimLightFPN-ECA-EMA	71.76	61.46	0.39	0.24	0.84	1

As can be observed from **Table 3**, among the various improved models, the YOLO-FastestV2-SimLightFPN-ECA-EMA model had the highest GPI of 0.84, the second-ranked model was the YOLO-FastestV2-SimLightFPN-ECA-EMA model with a GPI of 0.84, and the third-ranked model was the YOLO-FastestV2-ECA-EMA model with a GPI of 0.81. The highest accuracy of the YOLO-FastestV2-SimLightFPN-ECA-EMA model may be because it combines both the ECA and EMA attention mechanisms as well as the SimLightFPN module, which may have made the model more efficient in feature extraction and fusion. Although the GPI values of YOLO-FastestV2-SimLightFPN-LSKA-EMA and YOLO-FastestV2-SimLightFPN-ECA-EMA are close to each other, YOLO-FastestV2-SimLightFPN-LSKA-EMA performed better in other performance metrics (e.g., mAP@0.5, and mAP@.5:.95) is better, which suggests that it has an advantage in terms of accuracy. Compared with the YOLO-FastestV2-SimLightFPN-ECA-EMA model, the YOLO-FastestV2-SimLightFPN-LSKA-EMA model has a higher computational complexity and number of parameters, which may lead to limitations in its deployment performance on mobile platforms. The YOLO-FastestV2-ECA-EMA model had a slightly lower GPI than the other two models, but it also performed well in terms of accuracy. The most efficient model, YOLO-FastestV2-SimLightFPN-ECA-EMA, achieved a high level of accuracy while maintaining low computational requirements.

## 4 Conclusion

Accurate detection and measurement of the number of wheat spikes are important for the rapid prognosis of wheat yield and national food security. This study is based on fusing images of wheat spikes from multiple datasets to construct a model for wheat spike detection that can be used for preliminary estimation of wheat yield on mobile devices. The accuracy and reliability of the model were assessed by analyzing the differences between the model detection results and actual labeling results. In this study, the YOLO-FastestV2 model was used as the basis for constructing a wheat spike detection model. To improve wheat spike detection the accuracy, three attention mechanisms (ECA, EMA, and LSKA) were introduced and combined with YOLO-FastestV2 to construct a new model. In this study, the attention mechanism was added after the base and output phases of the backbone network. The YOLO-FastestV2-ECA1 model with ECA attention added after the base phase demonstrated the highest improvement in accuracy (P=82.64%, R=77.29%, AP=79.96%, and F1 = 79.88%). Conversely, the YOLO-FastestV2-LSKA2 model with the LSKA attention mechanism added after the output phase exhibited the highest accuracy gains (P=82.95%, R=77.01%, AP=80.06%, and F1 = 79.87%). To further enhance the extraction performance of target features in the backbone network, this study selected the ECA and LSKA attention mechanisms to improve the accuracy of the model after the base phase and the LSKA and EMA attention mechanisms that improved the accuracy of the model after the output phase to construct a model with a two-stage increase in the attention mechanism. The YOLO-FastestV2-ECA-EMA model demonstrated the highest accuracy (P=83.84%, R=78.11%, AP=81.42%, and F1 = 80.88%) among the two-stage models for increasing the attentional mechanisms. In this study, SimLightFPN was proposed by introducing SimConv to simplify and improve the feature fusion capability of the LightFPN module compared with the unimproved YOLO-FastestV2 model. This resulted in an increase of 2.84% in P, 2.19 in R, 2.81% in AP, and 2.5% in F1, compared with the original model. We then constructed a hybrid wheat spike detection model by combining a two-stage model with simultaneous addition of attention mechanisms and SimLightFPN. Among the hybrid models, the YOLO-FastestV2-SimLightFPN-LSKA-EMA model exhibited the highest accuracy (P=84.28%, R=78.37%, AP=81.85%, and F1 = 81.22%). In the comprehensive evaluation of the models, the GPI value of YOLO-FastestV2-SimLightFPN-ECA-EMA was ranked No. 1 (0.84), which is the optimal model under the combined consideration of model accuracy and model complexity. Currently, we have only used one lightweight machine-learning model for improvement and evaluation, and we will explore more machine-learning models in our future research and combine different network structures and techniques to achieve a wider range of applications on diverse datasets.

## Data availability statement

The raw data supporting the conclusions of this article will be made available by the authors, without undue reservation.

## Author contributions

SQ: Conceptualization, Methodology, Software, Writing – original draft. ZQ: Data curation, Formal analysis, Supervision, Writing – review & editing. WW: Software, Visualization, Writing – review & editing. FW: Formal analysis, Writing – review & editing. XJ: Funding acquisition, Investigation, Writing – review & editing. JJ: Funding acquisition, Visualization, Writing – review & editing. LZ: Funding acquisition, Software, Writing – review & editing. YS: Funding acquisition, Investigation, Writing – review & editing.
